# DNA Self-Assembly: From Chirality to Evolution

**DOI:** 10.3390/ijms14048252

**Published:** 2013-04-15

**Authors:** Youri Timsit

**Affiliations:** Centre National de la Recherche Scientifique, Aix-Marseille Université, IGS UMR7256, Marseille 13288, France; E-Mail: youri.timsit@igs.cnrs-mrs.fr; Tel.: +33-4-91-82-54-27

**Keywords:** chromatin, topology, higher-order structures, topoisomerase, crossover, DNA packaging

## Abstract

Transient or long-term DNA self-assembly participates in essential genetic functions. The present review focuses on tight DNA-DNA interactions that have recently been found to play important roles in both controlling DNA higher-order structures and their topology. Due to their chirality, double helices are tightly packed into stable right-handed crossovers. Simple packing rules that are imposed by DNA geometry and sequence dictate the overall architecture of higher order DNA structures. Close DNA-DNA interactions also provide the missing link between local interactions and DNA topology, thus explaining how type II DNA topoisomerases may sense locally the global topology. Finally this paper proposes that through its influence on DNA self-assembled structures, DNA chirality played a critical role during the early steps of evolution.

## 1. Introduction

Transient or long-term DNA self-assembly participates in essential genetic functions. For example, the compaction and the three-dimensional organisation of DNA are crucial for cellular processes such as transcription, DNA replication and segregation of daughter chromosomes during cell division. Also, bringing DNA sites into proximity is required for DNA recombination, chromatin packaging and building architectural complexes that control transcription and replication [1–3]. Although short-range contacts between double helices have been considered to be strongly repulsive, DNA is condensed under various conditions of condensing agents, cations or polyamines and may form organized phases or DNA liquid crystals [4,5]. In such arrangements, arrays of parallel stacks of helices are formed and since the inter-axial distances between double helical segments is about 25–32 Å, the DNA duplexes do not form direct intermolecular interactions [6,7]. In these conditions, the parallel packing of helices is only moderately influenced by the helical nature of DNA and its sequence [8–10].

In contrast, the closely packed DNA helices observed in DNA crystals are profoundly influenced by the DNA geometry, chirality and sequence [11–13]. The formation of tight DNA crossovers allows the closest approach between double helices by both minimizing their electrostatic repulsion and optimizing the mutual docking of attractive complementary surfaces. Interestingly, recent theoretical and experimental studies have indicated that close DNA-DNA interactions can also occur in solution, in the presence of divalent cations [14–18]. Although largely overlooked, these interactions have been found to play important biological roles for both controlling the architecture of higher-order DNA structure and DNA topology [19–21]. The detailed knowledge of the structure and energetics of close DNA-DNA interactions is therefore indispensable for a complete understanding of these functions at the molecular level. The present review is focused on the properties of close and direct DNA-DNA interactions. It is shown here that direct contacts between helices greatly enhance the effect of chirality on the overall architecture of the higher-order structures. It is proposed here that the structural simplification of the genetic material in passing from the RNA to the DNA world has contributed to store and pack larger genomes, in favouring inter-helical interactions governed by simple rules that are compatible with the codified hierarchical assembly of double helices at multiple levels.

## 2. From Double-Helix Chirality to Stable DNA Self-Fitting

### 2.1. Ubiquitous Crossovers

**Figure 1:**
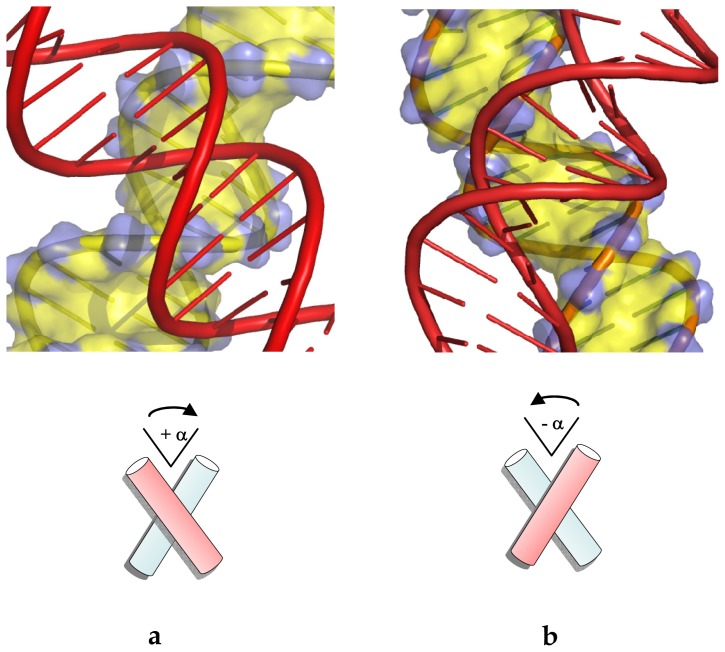
Chiral B-DNA crossovers. (**a**) Right-handed crossover assembled by the mutual fit of the backbones into the major-groove; (**b**) Left-handed crossover assembled by major groove-major groove interaction.

More than twenty years ago, crystallographic studies revealed that B-DNA helices can self-assemble into tight right-handed DNA crosses by the mutual fit of their sugar-phosphate backbone into the major groove, thus challenging the concept that the DNA-DNA interactions are repulsive (Figure 1a) [22]. These spectacular structures have been called *right-handed crossovers* since they are characterized by positive values of their crossing angle. Since the sugar-phosphate backbone is fitted into the major groove, the crossing angle is dictated by the chirality and geometry of the double helix—mainly the angle between the major groove and the helical axis. Thus, the self-fitting of double helices into a crossed structure both avoids electrostatic repulsion between double helices and optimizes energetically favourable intermolecular contacts [23,24]. Remarkably, most of the B-DNA right-handed crosses examined to date are assembled by the major groove-backbone interaction, involve cytosine-phosphate group interaction (see below) and are stabilized by divalent cations. In contrast, the A-form double helices, in both DNA or RNA preferentially self-assemble into right-handed crossovers formed by minor-groove backbone interactions. Indeed, in A-form double helices, this is the shallow minor groove of the A-form that is devoted to intermolecular interactions [25]. Interestingly, one of the most common elements of the ribosome structure is the interaction of RNA double helices via minor grooves [26]. Inter-helical packing involving minor-groove backbone interactions has been observed in the crystal packing of many RNA oligonucleotides [27,28]. Moreover, this so called “along-groove” packing motif that has been also observed within the structure of the 23S RNA of the large ribosomal subunit is thought to play a role in ribosomal function such as tRNA translocation [29,30]. The role of the DNA sequences is also different in the packing of A- and B-DNA helices. Indeed, a comparison of DNA crystal packing modes revealed that the interactions between A-DNA helices are much less dependent of the DNA sequence than the B-DNA ones [12]. Probably because the shallow minor groove of the A-form provides the opportunity to form many Van der Waals and hydrophobic interactions, their stable association has been found less dependent of the formation of specific hydrogen bonds. In contrast, the tight association of B-DNA helices is greatly influenced by the DNA sequence.

Left-handed B-DNA crossovers that are characterized by negative values (−40° and −80°) of the crossing angles have been also observed in many RNA or DNA crystals. However, this mode of assembly in which the helices are juxtaposed by groove-groove interactions, prevents the tight self-fitting of the double helices and is neither stabilized by direct sequence-specific contacts between DNA segments, nor by intermolecular divalent cation bridges [31] (Figure 1b).

### 2.2. Cytosine and DNA Self-Assembly

**Figure 2:**
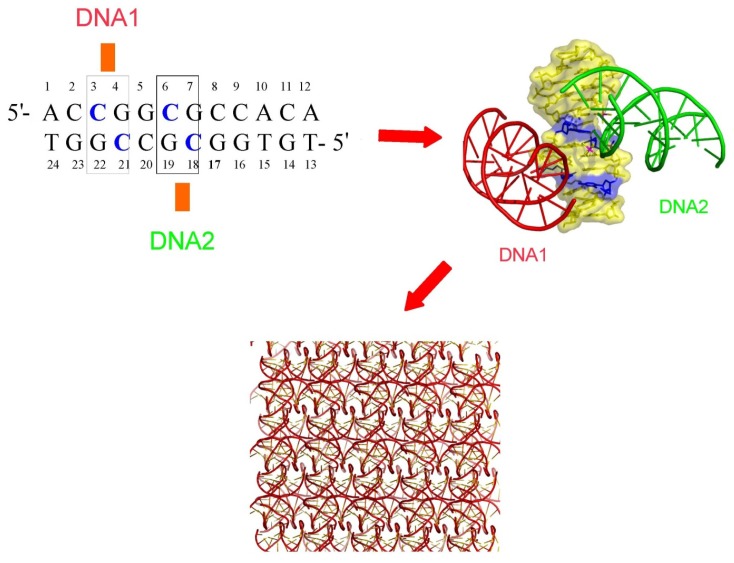
Design of crystal lattice from DNA sequence. The cytosines (represented in blue) along the duplex sequence define the anchoring points for groove-backbone interaction. The crossing of the duplexes within the crystal lattice is therefore controlled by the DNA sequence.

Right-handed crossovers of B-form DNA double helices are therefore unique in that they are assembled by a sequence-dependent interaction. Crystallographic studies have shown that while the B-DNA double helix dictates the geometry of inter-helical assembly, cytosines play a key role in controlling the interaction through specific interaction of their N4 amino groups with phosphate groups [22–24]. These studies provided the basic principles for designing DNA sequence that control the precise organization of double helices into a 3D lattice (Figure 2).

Thus, the formation of inter or intramolecular H-bonds between the N4 amino group of cytosine and a phosphate group plays a key role in controlling DNA-DNA interactions in a sequence dependent manner.

**Figure 3:**
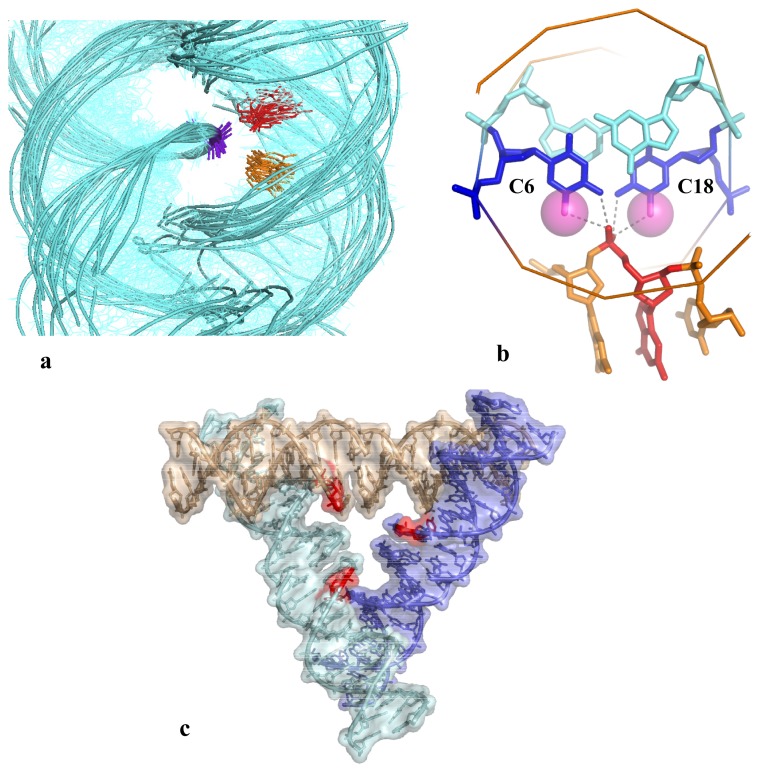
Cytosine and DNA self-assembly. (**a**) Superimposed self-fitted right-handed DNA crossovers found in the Nucleic Acids Database. The cytosines involved in the interaction are represented in red and orange; (**b**) 5-Methyl cytosine stabilizes the groove-backbone interaction through the formation of C–H…O interactions with the phosphate group; (**c**) The role of the sequence in DNA self-assembly: the cytosines (represented in red) dictate the organisation of the triangular motifs.

Consistent with these findings, a recent survey of the Nucleic Acids Database shows that, without exception, cytosine-phosphate interactions are strictly required for stabilising right-handed DNA crossovers (Figure 3a). Probably due to the vicinity of the N7 group that displays a negative potential, the N6 amino group of adenine has not been found to substitute the N4 amino group of cytosine for this type of interaction [20]. This finding is consistent with other studies showing that the major groove edge of cytosines has a positive potential [32,33].

**Figure 4:**
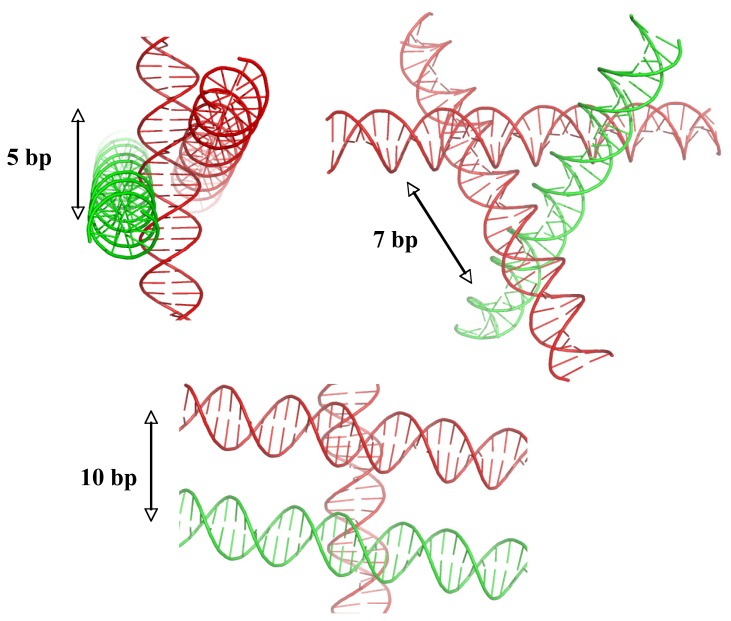
DNA supramolecular construction set. The double helix imposes discrete geometric solutions for its 3D assembly into simple motifs. Representation of packing motifs formed by two helices crossing a third one, at anchoring points separated by 5 bp, 7 bp and 10 bp, respectively. An equilateral triangle is formed when the intersection points of three DNA segments are 7 bp distant.

In addition, crystallographic studies of methylated DNA duplexes showed that C5-methyl cytosines also promote and stabilize the formation of DNA crossovers at the modified C5-mpG sequences [34]. The two methyl groups form a hydrophobic clamp which traps the incoming phosphate through C-H…O interactions that further stabilize the helical assembly (Figure 3b). Our method for designing DNA crystals has been used successfully for the systematic crystallisation of DNA molecules of various sequences and sizes [12] as for example, the spectacular DNA triangular motifs of DNA dodecamer and decamer duplexes [22–24] (Figure 3c). This analysis also revealed how the double helix dictates simple geometric rules for its spatial assembly. For example, Figure 4 shows how the relative orientation of two DNA helices may be controlled by the spacing of their anchoring points along a third one (Figure 4).

The DNA triangles recently designed by Zheng *et al.* (2009) [35] were conceived according to our conceptual and methodological framework. Indeed, the architecture of these triangles corresponds exactly to the triangular motif observed in the crystal packing of a decamer duplex crystallized in 1994, thus following the rules imposed by DNA self-fitting [19,24]. They correspond to a unique geometrical solution in which three DNA segments cross each other at a distance of 7bp (Figure 4b). The general applicability of these principles has subsequently been supported by the crystal structure of 4-way junctions whose stability also depends on cytosines placed at specific positions [36,37]. Overall, these works have thrown light on the particular role of cytosine bases for controlling spatial organisation and the stability of tertiary DNA assemblies.

### 2.3. Differential Stability of Chiral Crossovers

The free energy of interactions of DNA duplexes in right and left-handed crossovers as a function of divalent cation concentration in solution has been investigated using molecular dynamic simulations [18]. This study showed that right-handed DNA crossovers (Figure 1a) are thermodynamically stable in solution in the presence of divalent cations. Consistent with recent theoretical and experimental observations of close DNA-DNA interactions in the presence of divalent cations [14–17], a short-range attraction of about −4 kcal·mol^−1^ between the self-fitted duplexes was predicted in the presence of divalent cations [18]. Attractive forces at short-range stabilize the DNA-DNA association with inter-axial separation of helices less than 20 Å. Right-handed crossovers, however, dissociate in the presence of monovalent ions only. In solution, the acute angle, by which the two B-DNA duplexes cross one another in the right-handed geometry, fluctuates around an average value of 84° ± 6°, a value close to that observed in the R3 crystal packing [23]. The tight spread around this angle confirmed that the major groove induces a strict geometric constraint on the mutually fitted structures [24]. Also, consistent with the crystallographic studies, molecular dynamic simulation showed that two helices remain assembled by specific cytosine-phosphate interactions and bridging Mg^2+^ ions at the duplex interface. The repulsion of the negatively charged backbone is circumvented both by the specific relative orientation of helices and by the presence of Mg^2+^. Therefore, similar structural features of the right-handed crossovers are present in solution and in the crystal environment. Moreover, simulated DNA triangles constructed from 20-mer sequences are also stabilized by similar interactions in solution. In contrast, left-handed crossovers are unstable at similar ionic conditions and result in a swift dissociation of the helices. Without specific intermolecular interactions, left-handed helix juxtapositions by major groove-major groove interaction (Figure 1b) are stable only in the crystallographic environment but appear to be unstable in solution.

### 2.4. Role of Divalent Cations in DNA Assembly

Molecular dynamic simulation studies have also shown that the stabilisation of right-handed crossover increases as the Mg^2+^/duplex stoichiometric ratio increases [18]. A minimum of 8 Mg^2+^ per duplex is required to keep the duplexes anchored together with an associated binding free energy of about −4 kcal·mol^−1^. Higher Mg^2+^ concentrations (16 Mg^2+^/duplex) strengthen the helical interaction further and increase the associated binding free energy to −7 kcal·mol^−1^. At lower Mg^2+^ concentrations (4 Mg^2+^/duplex) no net attraction was visible. However, monovalent ions cannot replace the effect of Mg^2+^ to induce attraction between DNA helices even at high Na^+^ concentration (56 Na^+^/duplex). Importantly, during the simulation, Mg^2+^ ions occupied the divalent cation binding sites observed in the crystal structure of self-fitted duplexes. In contrast, no specific Mg^2+^ binding site was observed in the control simulations of isolated duplexes and hence we suggest that specific binding sites are formed simultaneously with the formation of the crossover structure. These data are consistent with the crystallographic studies that showed that the diffraction power of crystals of duplexes assembled via groove-backbone interactions was strictly correlated with the Mg^2+^/duplex stoichiometric ratio. Best diffracting crystals were obtained with 16 Mg^2+^ per oligonucleotide while very large crystals obtained with 1 Mg^2+^ per oligonucleotide did not diffract at all [22].

The strict requirement for Mg^2+^ to stabilize tight DNA-DNA interactions is also consistent with recent experimental and theoretical data. For example, SAXS and light scattering experiments indicated DNA-DNA repulsion in the presence of monovalent ions (up to [Na^+^] of 600 mM) but increasing attraction above [Mg^2+^] of 50 mM [14]. Also, recent theoretical work that used the tightly bound ion model found that helices repel one other in the presence of monovalent ions while divalent cations are able to induce attraction between two DNA helices [15]. These findings have been supported more recently by AFM studies on supercoiled DNA [38]. In addition, a recent study has also reported that DNA duplexes can self-assemble at nanomolar DNA concentrations in the presence of Mg^2+^[16]. Divalent cations and in particular Mg^2+^ ions are also required for the folding of both DNA and RNA molecules. They mediate the folding of Holliday junctions from a planar open structure into a compact stacked conformation [39]. Indeed, four-way junctions and right-handed crosses share an analogous geometry that is stabilized by similar tertiary interactions involving cytosines and Mg^2+^[23,34]. The folding of particular RNA motifs found in many functional RNA molecules also requires specific divalent cations [40–43]. A common feature in most of these structures is the anchoring of a phosphate group to a guanine base through a divalent cation bridge. Thus, among all cations available in physiological conditions, divalent cations have the unique property of stabilizing specific and tight intra- and intermolecular interactions between nucleic acid segments by forming guanine-phosphate bridges. In contrast, monovalent ions that are more diffuse around DNA and RNA may have an important role in the long-range steering of duplexes, as for example in the parallel alignment of double helices found in liquid crystals [5,13].

### 2.5. DNA Chirality and Sequence Controls the Architecture of Higher-Order DNA Structures

Like a directing piece for a “supramolecular construction set”, the B-DNA double helix dictates the overall geometry of DNA self-fitted assemblies. As exemplified in Figure 4, its periodic structure and sequence impose basic geometric constraints that restrict the spatial organization of DNA segments and dictates the architecture of simple and elementary structural DNA motifs (see above, Section 2.2). The DNA sequence encodes specific signals for positioning intra- or intermolecular DNA-DNA interactions: cytosine and guanine act conjointly to define the emplacement of DNA-DNA crossing by either establishing direct or cation-mediated intermolecular DNA-DNA interactions, respectively. Conversely, it has been found AT rich regions are less suitable for tight DNA-DNA interactions [20]. These findings may therefore contribute to the understanding of how local forces contribute to the organisation of DNA higher-order structures such as, for example, the compaction of the 30 nm chromatin fibre [44,45]. Indeed, it is well established that electrostatic forces govern primarily the folding of the chromatin fibre [46] and the strong dependence of chromatin compaction on cation binding indicates that chromatin folding involves close DNA-DNA interactions. Although recent experimental data support a compact interdigitated solenoidal structure, the exact mode of organisation of nucleosomes and linker DNA within the chromatin still remains a matter of controversy [47]. However, whatever its exact mode of assembly, experimental studies converge to indicate that chromatin folding involves close interactions between the linker DNA and/or between the nucleosomal DNA.

**Figure 5:**
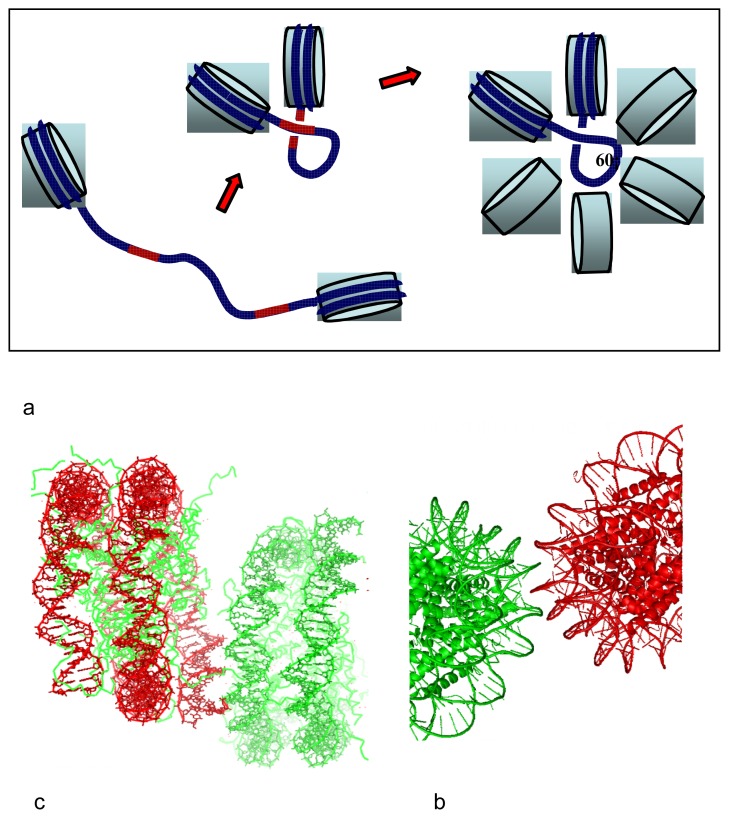
DNA-DNA interactions control the assembly of the chromatin fibre. (**a**) The model proposes that DNA geometry and sequence contribute to organise tighly packed regions in chromatin. The GC sequences suitable to form tight DNA-DNA interactions are represented in red. The geometric constraints imposed by the groove-backbone interaction influence the overall architecture of the fibre; (**b**) and (**c**) Right-handed crossovers between nucleosomal DNA observed in their crystal structure of [47] and [48], respectively.

In this context, the recent discovery that right-handed crossovers are stable at close to physiological conditions [18] supports the idea that close DNA-DNA interactions contribute to organize the nucleosomal or linker assembly within the chromatin fibre as described in Figure 5a[23,24]. In this model, while the double helix geometry controls the overall geometry of the fibre, sequence patterns promote close DNA-DNA associations in specific regions, between DNA linkers or nucleosomal DNA (Figure 5a).

Our model proposes that the cell may dispose of a collection of DNA-DNA interactions with varying degree of stability that can be exploited for tuning chromatin compaction. In addition, the finding that 5-methyl cytosine promotes close DNA-DNA interactions [34] may also explain how DNA methylation contributes to compact the chromatin fibre. Consistent with these hypotheses, nucleomes have been observed to spontaneously self-assemble by groove-backbone interactions in their crystal packing [47,48] (Figure 5b,c) and close DNA-DNA interactions are seen in the recent all-atom model of the chromatin fiber [49].

## 3. From Local DNA-DNA Interactions to Global DNA Topology

Transient DNA-DNA close interactions also play a critical role in the control of DNA topology. Indeed, within the interwound plectonemic supercoiled DNA, tight intersegmental contacts occur in the presence of divalent cations, under physiological conditions [38,50]. The importance of such close contacts has been also noted for the knotting of supercoiled DNA [51]. DNA supercoiling participates in essential cellular processes in both prokaryotes and eukaryotes, such as remote gene regulation, site-specific recombination and DNA replication [52,53]. While DNA is mainly negatively supercoiled in mesophilic cells, transcription and DNA replication may generate domains of positively supercoiled DNA *in vivo*[54–56]. Type II topoisomerases regulate the topological state of DNA by catalyzing the double strand passage reaction. These enzymes that are crucial in maintaining the fine balance of superhelical density also play a major cellular role in disentangling sister chromatids during replication [52].

Although acting locally, a remarkable property of type II topoisomerases is their sense of the global DNA topology. For example, when a negatively supercoiled ring is singly linked to a nicked ring, these enzymes preferentially unlink the ring rather than remove the supercoils [57]. Topoisomerases II can also simplify DNA topology and reduce the fraction of knotted or catenated circular DNA molecules well below thermodynamic equilibrium values [58]. They are also capable of chiral discriminiation between knots of opposite sign [59] and some of them such as DNA gyrase, topoisomerase IV and human topoisomerase IIα can discern the sign of supercoiled DNA in acting preferentially on positive supercoiled DNA [60–64]. Due to this striking ability to sense the global properties of DNA from local interactions, they have been compared to Maxwell’s demons [65]. How can topoisomerases discriminate between the different global topologies of a much larger DNA? Several hypotheses have been postulated to explain this phenomenon. For example, the protein induces a sharp bend in DNA at the binding site that provides unidirectional strand passage [58,66]. A kinetic proof reading model that requires two separate topoisomerase-DNA collisions for segment passage has been also proposed [67]. Alternatively, it has been suggested that the topological information may be embodied in the local geometry of DNA crossings and that topoisomerases act at the hooked juxtapositions of the strands [68,69]. Indeed the interplay of local and global properties constitutes a key element in the cellular function of DNA and local intra- or intermolecular DNA-DNA interactions play a central role by establishing a link between the two hierarchical levels of structural organization in DNA [9]. Recent experimental studies supported this view in showing that topoisomerase IV and DNA gyrase discriminate the sign of supercoiled DNA on the basis of the geometry of the DNA crossovers [60–63,70]. This puzzle has been made more complex recently by the finding that topoisomerase IV is highly processive on (+) supercoiled DNA and perfectly distributive on (−) supercoiled DNA [71]. How DNA crossover geometry influences the differential mobility of type II topoisomerases on supercoiled DNA of opposite sign represents therefore an interesting conceptual challenge. Knowing that type II topoisomerases recognise and act on DNA crossovers [72], the question can be formulated as: how do type II topoismerases sense the global DNA topology from the local geometry of DNA crossovers?

**Figure 6:**
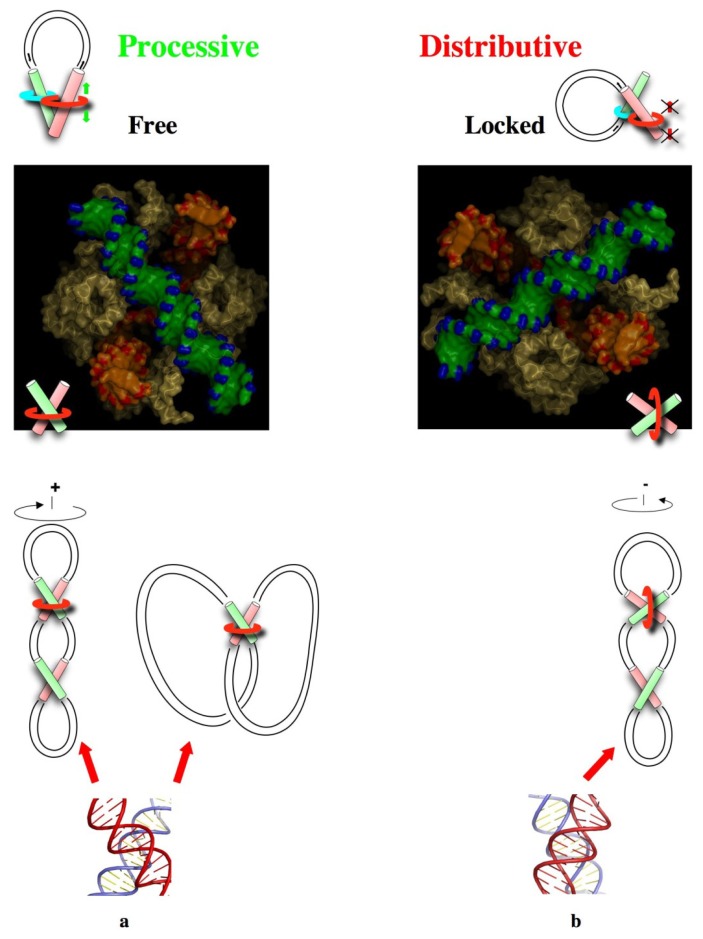
From DNA chirality to the local sensing of global topology. (**a**) Stable right-handed crossovers form spontaneously in relaxed, (+) supercoiled DNA or catenanes. They constitute the natural substrate of type II topoisomerases that clamp them across their large angle (middle). Thus, the topoisomerase ring does not form a topological link with the (+) supercoiled ring. The strand passage reaction generates a free configuration (top) that is consistent with the enzyme processivity; (**b**) Unstable left-handed crossovers are exclusively formed in (−) supercoiled DNA. Our model predicts that type II topoisomerases slightly deform and clamp them, across their large angle (middle). This imposes a different orientation in which the enzyme ring and the (−) supercoiled DNA ring are topologically linked. The strand passage reaction generates a locked configuration (top) that explains the distributive behaviour of the enzyme. The gate DNA-segment (G) and transported DNA-segment (T) are represented in red and green, respectively.

Applying our crystallographic lessons to DNA topology brought two useful insights to solve this question. First, it has been demonstrated that the various topological states of the cell are associated with different local inter-segmental interactions [19]. Knowing that right- and left-handed crossovers not only differ by their geometry but also by their stability contributed to solve the mechanism of chiral discrimination by type II topoisomerases. Although probably transiently stable, the right-handed DNA crossovers constitute the most probable structure of site juxtaposition in physiological conditions. Consequently they not only occur preferentially in (+) supercoiled DNA for geometrical reasons, but they are also preferentially formed in the absence of superhelical stress, as in relaxed DNA, catenanes (two linked DNA rings) or loose knots for electrostatic reasons (Figure 6a). In contrast, the unstable left-handed crossovers are exclusively formed in negatively supercoiled DNA (Figure 6b).

Stable right-handed crossovers constitute therefore the local signature of an unusual topological state in the cell, such as positively supercoiled or relaxed DNA. Local information such as the differential stability and geometry of crossovers may be therefore exploited for sensing the global topology of DNA [19]. This suggested that type II topoisomerases discriminate (−) supercoiling from other topological states in preferentially acting on stable right-handed crossovers.

Second, a recent study has demonstrated how binding right-handed crossovers specifically across their large angle imposes a different topological link between the type II topoisomerases and the plectonemes of opposite sign (Figure 6, top) [21]. Within (+) supercoiled DNA, the enzyme ring is perpendicular to the long supercoiled DNA axis and is not topologically linked to the circular DNA chain. In (−) supercoiled DNA, the enzyme ring that is parallel to the supercoiled DNA axis forms an interlocked knot and is topologically linked with the circular DNA. The different topological links affect the enzyme freedom of motion and processivity and provide an explanation for the chiral discrimination [21].

## 4. DNA Self-Assembly and Evolution

DNA topology and topoisomerases have been the subject of adaptive pressure in organisms that live at different temperatures for maintaining the balance between the melting potential and functional stability [73]. The dynamics of plectonemic DNA supercoiling also plays a critical role in promoting interactions between remote sites in processes such as transcription initiation and site-specific recombination [53]. Indeed, several studies have shown that some particular local inter-segmental contacts alter the functional dynamic of supercoiled DNA [9]. Similarly, divalent cations that promote formation of stacked 4-way junctions [39] considerably slow down the kinetics of spontaneous branch migration [74]. Consequently, these dynamic properties should be also finely tuned as a function of the temperature and other physico-chemical parameters. It has been proposed that, chiral properties of DNA crossovers and the resulting asymmetrical behaviour of supercoiled DNA of opposite signs have contributed to orient early choices for DNA topology in the nascent DNA world [19].

Thus, among other physical properties of DNA, such as its anisotropic flexibility [75,76], or the fact that DNA is more easily untwisted than overtwisted, it is likely that the differential stability of chiral crossovers has influenced the choice of DNA topology in organsims that live at moderate temperature. In particular, the spontaneous formation of stable right-handed crossovers in relaxed or (+) supercoiled DNA may have posed challenges to mesophilic cells: in the presence of divalent cations, the stable inter-segmental interactions should make (+) supercoiled DNA significantly more “sticky” than (−) supercoiled DNA, along GC rich sequences. Indeed, from a functional point of view, right-handed DNA crosses can be viewed as a Janus-like DNA structure. While the stable inter-segmental interactions can be useful for closely packaging DNA into higher-order DNA structures, they may have a detrimental effect by impeding the global dynamics of the genome, if they occur without control within a plectonemic supercoiled DNA. It is therefore possible that these two opposite features may have led to different evolutionary strategies to adapt to mesophilic conditions where weak interactions that occur within right-handed crossovers can be expected to be stable.

Indeed, in contrast to life at high temperature that can tolerate various topological states of DNA—from negative supercoiled DNA to slightly positively supercoiled DNA [77–81], adaptation to mesophilic life is much more constraining on the topology of DNA: the genome of mesophilic organisms, including bacteria, archaea and eukarya, is systematically (−) supercoiled. All mesophilic bacteria have a DNA gyrase that introduce (−) supercoiling in a plectonemic form [73]. Particularly interesting is the case of mesophilic archaea. They have either acquired a gyrase that introduces negative supercoiling, or histones that wrap DNA into toroidal supercoils [73]. In other words, mesophilic organisms appear to have evolved to strictly avoid the presence of permanently relaxed or (+) supercoiled DNA in their genome. As mentioned above these topological states are expected to impede the dynamics of supercoiled DNA and affect functions. Maintaining permanent (−) supercoiling could therefore be viewed as preventing sticky interactions and promoting the “fluidity” required for various functions. Our model can also account for the observation that hyperthermophilic archaea tolerate other topological states of DNA, such as the relaxed or slightly (+) supercoiled states. Indeed, higher temperatures would decrease the stability of right-handed crossovers and restore the relative mobility of DNA segments. Second, wrapping DNA around histones in mesophilic archaea and eukarya can be viewed as an alternative mode of adaptation to the presence of sticky DNA-DNA interactions in their genome. It can be speculated that this regular mode of DNA packaging allows the organism to precisely control the position of right-handed crosses and to exploit their physical properties. As seen above, DNA self-association may contribute to stabilise the interactions between nucleosomes or DNA linkers within the chromatin fibre (Figure 5) [44].

**Figure 7:**
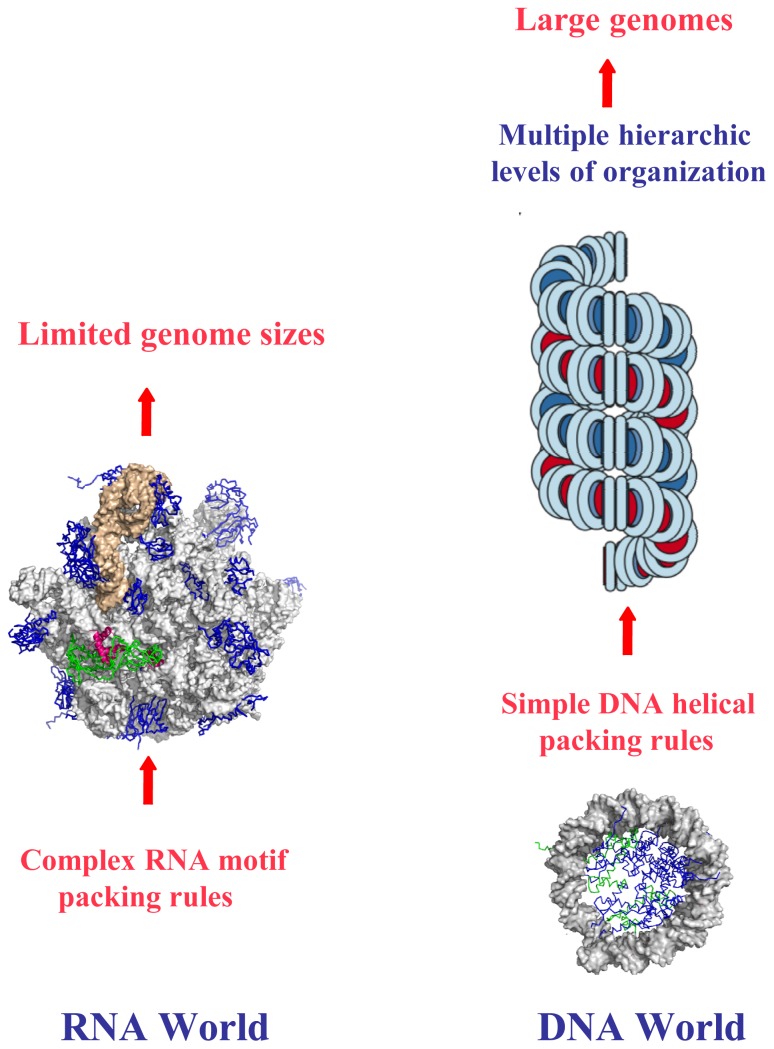
From chirality to genome evolution. Simple rules for DNA packaging allow multiple hierarchic levels of higher-order structure assembly.

On the other hand, emerging from the RNA world, the DNA has been selected to support the genetic information of complex organisms. It could be speculated that this early “choice” of the double helix has been influenced by its ability to self-assemble into a simple hierarchic organization. Indeed, RNA and DNA not only differ by their respective chemical stabilities, but also by their packing modes and consequently the overall architecture of their supramolecular assembly. Although similar electrostatic rules govern the self-contacts between RNA and DNA molecules, their helical interactions differ as a consequence of their distinct secondary structures. In addition, to adopt a regular A-conformation, the RNA molecules most often fold into more complex structural motifs due to the presence of the 2′-hydroxyl group and extended base pairing rules [82]. RNA structures are therefore characterized by flourishing modes of tertiary interactions, from simple inter-helical interactions to the docking of a wide repertoire of sequence-dependent 3D motifs [83,84]. The combination of these tertiary interactions may produce extremely complex structures such as a ribosome (Figure 7, left). In contrast, DNA molecules mainly form a regular B-DNA double helix that is only stabilized by canonical Watson-Crick base pairing and whose integrity is extremely sensitive to base pairing rules. Consequently, DNA world and its architecture are mainly directed by the reign of the double helix and its self-interactions. The evolutionary choice of austerity has therefore enhanced the role of chirality in the building of higher-order DNA structures. It is therefore likely that the use of simple and codified packing rules for DNA segments has been a determinant step for the evolution and packaging of large genomes of more complex organisms. Simple rules governing DNA-DNA interactions leave the possibility of adopting multiple hierarchic levels of organization, from the nucleosome to the mitotic chromosome (Figure 7, right). In contrast, to my knowlege, the ribosome that constitutes the ultimate level of organisation of RNA can only “pack” a few thousand RNA bases. Because of their fascinating structural diversity, the complexity and diversity of RNA motifs severely limit the potential to form higher levels of organisation. Thus, simplifying the self-assembled object may have been a key determinant during the early steps of evolution, for leaving the emerging life complexity.

## 5. Conclusions and Future Prospects

Crystal packing effects and crystal intermolecular contacts are most often considered as disturbing artefacts in crystallography whose main purpose is to solve the structure of a single molecule in a “structure-function” perspective. An alternative point of view is that crystal packing may mimic *in vivo* molecular interactions and bring precious insights about how a molecule is influenced by a defined intermolecular environment. Many crystal packings of nucleic acid double helices are indeed very similar to the dense packing of double helices observed in large nucleic acid assemblies such as the recently solved ribosomal particles or nucleosomal DNA. In the present review, an analysis of the reciprocal influence of the double helix structure on its crystal packing shows how the interplay of DNA sequence and electrostatic environment controls DNA-self assembly. This study reveals how tight DNA-DNA interactions greatly enhance the effect of chirality on the overall architecture of DNA higher-order structures thus providing the missing link for the local sensing of the global DNA topology by type II DNA topoisomerases. This study also suggests that the structural simplification of the genetic material in passing from the RNA to the DNA world has probably contributed to the storing and packing of larger genomes, in favouring the simple and codified hierarchical assembly of double helices. The detailed knowledge of the structure and energetics of close DNA-DNA interactions is therefore indispensable for a complete understanding of the genetic functions.

However, many questions remain to be solved as for example how do the B-A transitions affect the energetic and sequence dependence of tight DNA interactions? It has been proposed that the A-form of DNA protects the geometry of the active site of DNA-polymerases against deleterious structural variations of sequence hotspots, thus directly contributing to DNA-polymerase fidelity [85]. More recently, it has been suggested that the A-form of the G-segment found in the active site of type II topoisomerases, plays a similar role, in smoothing the minor groove surface in facilitating the fitting in a sequence-independent manner, of the incoming T-segment [21]. The presence of A-DNA in two key enzyme-DNA complexes such as DNA-polymerases and type II topoisomerases should be a stimulation to further examine the properties of B-A transitions and in particular, their effects on DNA compaction. Another question that has been recently rewiewed elsewhere [86] is how does DNA-DNA self-assembly influence the structure and dynamics of the double helix? Interstingly, tight DNA-DNA interactions have been reported to destabilize the double helix to various degrees, from the alteration of the base-stacking to the opening of the base-pairs, depending on the sequence. This has shown that DNA self-assembly provides a potential source of the energy required for DNA melting, thus suggesting a new molecular mechanism for strand separation and raising the challenging question: Is DNA a helicase? These findings should stimulate further characterization of the principles that govern DNA-directed base pair opening. Overall, these studies unveil that beyond being a passive molecular support of genetic information, DNA may actively participate in fundamental cellular functions.
